# High-Density Electroencephalogram Facilitates the Detection of Small Stimuli in Code-Modulated Visual Evoked Potential Brain–Computer Interfaces

**DOI:** 10.3390/s24113521

**Published:** 2024-05-30

**Authors:** Qingyu Sun, Shaojie Zhang, Guoya Dong, Weihua Pei, Xiaorong Gao, Yijun Wang

**Affiliations:** 1 Laboratory of Solid State Optoelectronics Information Technology, Institute of Semiconductors, Chinese Academy of Sciences, Beijing 100083, China; 2 School of Future Technology, University of Chinese Academy of Sciences, Beijing 100049, China; 3 Hebei Key Laboratory of Bioelectromagnetics and Neural Engineering, School of Health Sciences and Biomedical Engineering, Hebei University of Technology, Tianjin 300130, China; 4 Department of Biomedical Engineering, School of Medicine, Tsinghua University, Beijing 100084, China; 5 Chinese Institute for Brain Research, Beijing 102206, China

**Keywords:** brain–computer interface, code-modulated visual evoked potential, high-density EEG, small stimulus

## Abstract

In recent years, there has been a considerable amount of research on visual evoked potential (VEP)-based brain–computer interfaces (BCIs). However, it remains a big challenge to detect VEPs elicited by small visual stimuli. To address this challenge, this study employed a 256-electrode high-density electroencephalogram (EEG) cap with 66 electrodes in the parietal and occipital lobes to record EEG signals. An online BCI system based on code-modulated VEP (C-VEP) was designed and implemented with thirty targets modulated by a time-shifted binary pseudo-random sequence. A task-discriminant component analysis (TDCA) algorithm was employed for feature extraction and classification. The offline and online experiments were designed to assess EEG responses and classification performance for comparison across four different stimulus sizes at visual angles of 0.5°, 1°, 2°, and 3°. By optimizing the data length for each subject in the online experiment, information transfer rates (ITRs) of 126.48 ± 14.14 bits/min, 221.73 ± 15.69 bits/min, 258.39 ± 9.28 bits/min, and 266.40 ± 6.52 bits/min were achieved for 0.5°, 1°, 2°, and 3°, respectively. This study further compared the EEG features and classification performance of the 66-electrode layout from the 256-electrode EEG cap, the 32-electrode layout from the 128-electrode EEG cap, and the 21-electrode layout from the 64-electrode EEG cap, elucidating the pivotal importance of a higher electrode density in enhancing the performance of C-VEP BCI systems using small stimuli.

## 1. Introduction

The brain–computer interface (BCI) creates a direct information channel by connecting the brain to the external world [1]. The BCI system allows users to control external actuators in near real-time. For example, BCI applications based on electroencephalography (EEG) can be used to control computers or other devices to achieve prosthesis control [2], target detection [3], spelling [4], etc. Compared with invasive BCIs that implant electrodes into the brain, non-invasive BCIs based on scalp EEG measurement are safer, inexpensive, and easier to use [5]. Among various BCI paradigms, the visual evoked potential (VEP)-based BCI has attracted increasing attention in recent years [6].

Code-modulated VEP (C-VEP) BCIs encode each target using a pseudo-random sequence, similar to the code-division multiple access method in tele-communication. When a subject fixates on one of the stimuli, the stimulus can be identified by detecting C-VEP signals in the occipital lobe using template matching [7]. The M-sequence is the most widely used pseudo-random sequence, characterized by an autocorrelation function that is very close to a unit impulse function, and it is almost orthogonal to its time-shifted sequences [8]. In recent years, the C-VEP BCI has found widespread applications due to its ability to hit more targets and increase the information transfer rate (ITR) with a short calibration time [9]. Bin et al. successfully achieved an ITR of 108 bits/min with a 32-target paradigm [10]. Liu et al. employed multiple M-sequences for encoding, achieving 64 targets with an ITR of 184.6 bits/min [11]. Sun et al. utilized four gold sequences to hit 120 targets with an ITR of 265.74 bits/min [12].

VEPs evoked by stimuli in the central visual field surpass those evoked by stimuli in the peripheral field [13,14], thus allowing the control of the BCI through individuals’ gaze. In VEP-based BCI systems, the amplitude of EEG response highly depends on the size of the stimulus area. In addition to the decrease in VEP amplitude, the gaze target stimulus will face more interference from peripheral target stimuli when using a smaller stimulus size or a higher stimulus density, thus diminishing the performance of the BCI system [15,16,17,18]. In previous studies, the stimulus typically covered the majority of the central 4° of the visual angle, across the entire central fovea [18,19]. Zhang et al. investigated SSVEP responses within the subjects’ visual field, indicating that the optimal visual flicker arrangement for enhancing SSVEP is circular stimuli within a spatial distance of 4–6° [15]. Wei et al. investigated the impact of stimulus size and proximity on target identification in C-VEP BCI systems, recommending a stimulus size of 3.8° and a stimulus center-to-center distance of 4.8° [17]. Currently, there is very limited research on small and closely spaced VEP stimuli. For real applications, smaller stimulus sizes and closely spaced visual stimuli can enhance the spatial resolution of fixation, reduce the amplitude of eye movement in gaze control, reduce the occupied stimulus area, and improve the usability of BCI systems.

High-density EEG might provide more spatial information for detecting VEPs evoked by small stimuli. The widely used nine-channel electrode layout in the occipital and parietal areas may not be sufficient to capture all information concerning the VEP [6]. Currently, an inter-electrode distance of 2 cm is a commonly used and widely adopted configuration. However, multiple studies have suggested that reducing the inter-electrode distance to below 2 cm is beneficial, as it can provide additional neural information [20,21,22,23,24]. Employing a novel classification paradigm, Petrov et al. found that ultra-high-density electrodes with 1 cm inter-electrode separations could provide twice the signal-to-noise ratio for classifying visual brain responses elicited in bilingual contexts compared to high-density electrodes with 2–4 cm inter-electrode separations [23]. Song et al. indicated that high-density electrodes could achieve lower EEG signal reconstruction errors and enable more accurate source localization of EEG data [20]. Robinson et al. showed that high-density electrodes classified visual stimuli more accurately and could capture more spatial information, which provided enhanced insights into the spatio-temporal aspects of early visual processing [24].

In this study, C-VEP signals were recorded using a 256-electrode high-density EEG cap fitted with 66 electrodes over the occipital and parietal areas, with the inter-electrode distance set at 1.8 cm. For the stimuli of 0.5°, 1°, 2°, and 3°, offline and online experiments were performed. Data lengths used by each subject for each stimulus size in the online experiment were determined based on data from the offline experiments. Ultimately, average ITRs of 126.48 ± 14.14 bits/min, 221.73 ± 15.69 bits/min, 258.39 ± 9.28 bits/min, and 266.40 ± 6.52 bits/min were obtained for the four stimulus sizes in the online experiment. Furthermore, this study compared classification performance levels across different electrode coverage areas and quantities by implementing electrode selection, thereby substantiating the crucial role of high-density EEG in detecting C-VEPs elicited by small stimuli.

## 2. Materials and Methods

### 2.1. Experimental Environment

#### 2.1.1. Subjects

Sixteen healthy subjects (including seven females, aged between 23 and 30 years) were enrolled in both the offline experiment and the online experiment. All subjects possessed either normal or corrected-to-normal vision. All the subjects provided their signatures on informed consent forms, which had been previously approved by the Institutional Review Board of Tsinghua University, under the approval number 20230058.

#### 2.1.2. Data Acquisition

**Figure 1:**
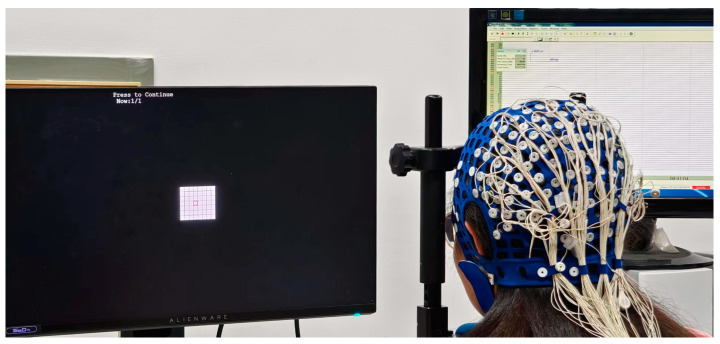
The experimental environment for the condition of 0.5° stimulus.

The experimental system consisted of a personal computer running on the Windows 10 operating system, accompanied by a Neuroscan Synamps2 EEG system (Compumedics Inc., Victoria, Australia). A 24.5-inch Alienware 2521H liquid crystal display (LCD) monitor, boasting a resolution of 1920 × 1080, was utilized for the display of stimuli at a refresh rate of 60 Hz. The experimental program was executed using Psychtoolbox version 3.017 on MATLAB 2022b (Mathworks Inc., Natick, MA, USA) [25]. Trigger signals for the stimulus events were transmitted from the computer to the EEG amplifier through a parallel port. During the experiment, the impedance of all electrodes was maintained at below 20 kΩ. The sampling rate for the raw EEG data was set at 1000 Hz, and a notch filter at 50 Hz was employed to eliminate electrical noise from power lines. The experimental environment with controlled brightness conditions was unshielded, as shown in Figure 1. Subjects were seated in a comfortable chair, with their heads resting on a chin rest to maintain a distance of 70 cm between their eyes and the screen.

**Figure 2:**
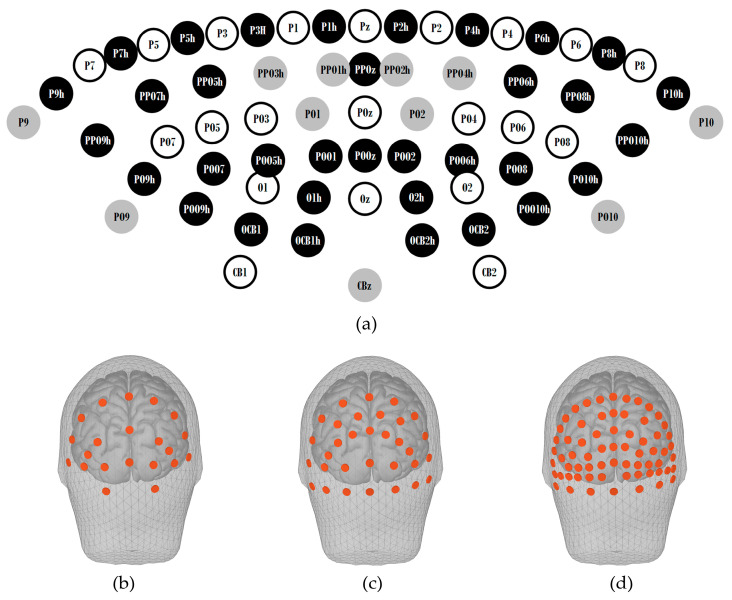
(**a**) The EEG electrodes used in this study. The electrodes with a black background are exclusive to the 66/256 layout, while those with a grey background are present in both the 32/128 layout and the 66/256 layout. The electrodes with a white background are concurrently found in the 21/64 layout, the 32/128 layout, and the 66/256 layout. The distribution of electrodes is then shown for the (**b**) 21/64 layout, (**c**) 32/128 layout, and (**d**) 66/256 layout.

A 256-electrode international 10/5 system EEG cap was utilized for the acquisition of C-VEP signals in this study. This cap was equipped with 66 Ag/AgCl electrodes placed over the occipital and parietal lobes, which is shown in Figure 2a (Pz, P1~P10, P1h~P10h, PPOz, PPO1h~PPO10h, POz, PO1~PO10, PO9h, PO10h, POOz, POO1, POO2, POO5h, POO6h, POO7, POO8, POO9h, POO10h, Oz, O1, O1h, O2, O2h, OCB1, OCB1h, OCB2, OCB2h, CBz, CB1, CB2). The reference electrode was positioned at the vertex. 

In this study, to highlight the benefits of high-density EEG, BCI performance levels when using 21 electrodes from the 64-electrode cap and 32 electrodes from the 128-electrode cap were compared with those when using 66 electrodes from the 256-electrode cap. For convenience, the electrode layouts mentioned above are denoted as 21/64, 32/128, and 66/256 layouts in the following text. The spatial distributions of electrodes across these three layouts are illustrated in Figure 2b–d, created using brainstorm [26]. The inter-electrode distance for the layout was determined by calculating the distances to the two nearest electrodes for each electrode, and then averaging these distances across all electrodes. The inter-electrode distances for the three layouts are 3.1 cm, 2.8 cm, and 1.8 cm, respectively.

### 2.2. Target Modulation

In this study, the stimuli were modulated using pseudo-random codes that possess good autocorrelation properties. Specifically, a 31-bit binary M-sequence and its temporally shifted sequences were employed to render the stimulus frames of 30 stimuli, with the duration of 0.52 s at a screen refresh rate of 60 Hz. The M-sequence used was ‘0011100110111110100010010101100’, where ‘0’ denotes a black frame (RGB: 0, 0, 0) and ‘1’ indicates a white frame (RGB: 255, 255, 255).

**Figure 3:**
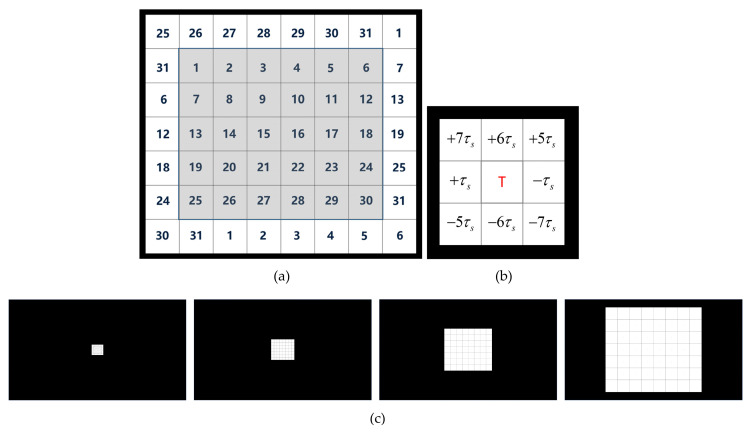
(**a**) The GUI of the experiment. Numbers represent temporally shifted lags for encoding, the shaded area represents 30 targets, and the white area represents 26 paddings. (**b**) An example of equivalent neighbors. The value in the target is the number of lags compared to the central target. (**c**) The relative size of the stimulus matrix on the screen. The sizes from left to right are 0.5°, 1°, 2°, and 3°, and the black area represents the background of the full screen.

The graphic user interface (GUI) of the experiment is shown in Figure 3a. The GUI contains 30 targets, arranged in a 5 × 6 matrix, which were modulated by a 31-bit M-sequence and its temporally shifted sequences with a lag of 1 bit. According to the principle of equivalent neighbors [10], 26 padding elements are strategically positioned around the perimeter of targets. An example of equivalent neighbors is illustrated in Figure 3b. For any given target, the lags for the top, bottom, left, and right positions are established at +6 bits, −6 bits, +1 bit, and −1 bit, respectively. In the experiment, 4 sizes of stimuli were used, 0.5°, 1°, 2°, and 3°, which are shown in Figure 3c. For each size, each target on the GUI was a square of 21, 42, 86, or 130 pixels in length and width. The interval between adjacent stimuli was 1 pixel in black.

### 2.3. Experimental Setup

In this study, four stimulus sizes (0.5°, 1°, 2°, 3°) were used; those and an example of the relative size of the stimulus matrix on the screen are shown in Figure 3c. The experiment for each stimulus size comprised an offline experiment and online experiment, with each stimulus size presented in a random sequence.

#### 2.3.1. Offline Experiment

The offline experiment includes two stages: training stage and testing stage. The training stage was aimed at acquiring training data across 4 stimulus sizes from a reference target, which was identified as the 15th target (see Figure 3a) in this study. At the start of the training stage, a GUI corresponding to the specific size was displayed at the center of the screen, and we initiated playback upon subjects pressing a key. Subjects were asked to concentrate on the reference target, which was cued by a 1-pixel red frame. Ten blocks were completed, wherein the 31-bit code was sequentially presented bit by bit across 12 stimulus cycles, each lasting 6.2 s. Subsequently, after the completion of each block, a 1 s rest period followed. 

The testing stage aimed to approximate the system performance under different stimulus durations using the individualized templates and spatio-temporal filters created in the training stage. It was conducted immediately following the training stage, and during the testing stage, a total of 3 blocks of experiments were conducted, with each block consisting of 30 trials. Within each block, the 30 targets were presented in random order for 1 trial. Upon completion of a block, subjects were instructed to take a rest period. Ultimately, data of 3 trials were obtained for each target. Each trial lasted 4.12 s, comprising 0.5 s for gaze shifting and 3.62 s of data acquisition. Throughout this period, targets were cued by a red frame with a width of 1 pixel. During the gaze shifting, the stimulus continued to flicker, with each cycle lasting 0.5 s corresponding to the 2 to 31 bits of the M-sequence. Following the completion of gaze shifting, the 31-bit code was repeated for 7 cycles, spanning a duration of 3.62 s. A trigger signal was dispatched at the start of each cycle, occurring at intervals of 0 s, 0.52 s, 1.03 s, 1.55 s, 2.07 s, 2.58 s, and 3.1 s. The cue for the next target was presented immediately following the end of the previous target and subjects were instructed to swiftly shift their gaze to the next target. When a test stage for a stimulus size was completed, the ITR was calculated to determine the stimulus duration used in the online experiment.

#### 2.3.2. Online Experiment

The online experiment, conducted following the offline experiment, reflects the performance of the online system under real-world application conditions. The stimulus duration varied for different subjects and different sizes of stimuli and was determined based on the testing stage of the offline experiment. This duration $t_{online}$ represents the stimulus duration that achieved the highest ITR in the testing stage of the offline experiment, ranging from 1 to 7 cycles (0.52 s to 3.62 s, with steps of 0.52 s). The individualized templates and spatio-temporal filters in the online experiment were created in the training stage of the offline experiment. During the online experiment, a total of 3 blocks of experiments were conducted, with each block consisting of 30 trials. Within each block, the 30 targets were presented in random order for 1 trial. Upon completion of a block, subjects were instructed to take a rest period. Ultimately, data of 3 trials were obtained for each target. Each trial lasted $\left(0.5+t_{online}\right)$ seconds, comprising 0.5 s for gaze shifting and $t_{online}$ seconds of data acquisition. Throughout this period, targets were cued by a red frame with a width of 1 pixel. During the gaze shifting, the stimulus continues to flicker, with each cycle lasting 0.5 s, corresponding to the 2 to 31 bits of the M sequence. The cue for the next target was presented immediately following the end of the previous target and subjects were instructed to swiftly shift their gaze to the next target. Once the calculation of the results for the previous trial was completed, immediate feedback was provided using a blue-colored frame with a width of 1 pixel.

### 2.4. Task-Discriminant Component Analysis

The task-discriminant component analysis (TDCA) is a discriminative model that utilizes discriminant analysis to seek out the spatio-temporal filter with respect to all classes. In SSVEP data processing, the augmented data are projected onto the subspace spanned by a sine–cosine reference signal [27]. However, in the case of C-VEP, this step is omitted due to the lack of a standardized reference signal. In this study, a modified TDCA algorithm was employed to classify the 30 targets for C-VEP.

In each training trial, the dimensionality of the EEG data is initially increased, as follows:

(1) $$\tilde{X}={\left[X^{T},X_{1}^{T},\cdots ,X_{l}^{T}\right]}^{T}$$

where $\tilde{X}\in R^{\left(l+1\right)N_{c}\times N_{p}}$ represents the augmented EEG trial, $X\in R^{N_{c}\times N_{p}}$ represents the training trial, $N_{c}$ denotes the number of channels, and $N_{p}$ is the data length. $X_{l}$ refers to the EEG trial delayed by l time points, encompassing the data copy from time (in data points) l+1 to $N_{p}+l$. Subsequently, the TDCA spatio-temporal filters are trained using the augmented data, as follows:

(2) $$\underset{W}{\max}imize\frac{tr\left[W^{T}H_{b}H_{b}^{T}W\right]}{tr\left[W^{T}H_{w}H_{w}^{T}W\right]}$$

where $H_{b}$ is the between-classes difference matrix and $H_{w}$ is the within-class difference matrix.

When obtaining augmented testing data, to ensure that data points exceeding $N_{p}$ are not included in the test, zero-padding is applied to any points beyond $N_{p}$ [27]. The template ${\bar{X}}_{k}$ is generated by averaging the labeled training data categorized under the kth class. The EEG data from the unlabeled target, represented as Y, together with the template ${\bar{X}}_{k}$, undergo spatial filtering to calculate their two-dimensional correlation coefficient:

(3) $$r_{k}=\rho ({\bar{X}}_{k}^{T}W,Y_{k}^{T}W)$$

The label of the target is identified as the one corresponding to the highest correlation coefficient $r_{k}$:

(4) $$\tau =\underset{k}{\arg\max}r_{k},k=1,2,\cdots ,N_{t}$$

In this study, the TDCA algorithm was configured with subspaces equal to the number of channels used and a delay lag of l=4. The activation patterns [28] were computed to depict the latent spatial patterns learned by the TDCA model. The activation patterns are defined by

(5) $$A=\sum_{x}W\sum_{\hat{S}}^{-1}$$

where W signifies the spatial filter, and the variables $\sum_{x}$ and $\sum_{\hat{S}}^{-1}$ represent the covariance matrices associated with the initial EEG recordings and the estimated source EEG, respectively. For the analysis of activation patterns in TDCA, the EEG data were re-referenced using a common average reference, and activation patterns were computed using a delay lag of 0. These activation patterns were averaged across subjects and normalized using values from the Oz channel [27].

### 2.5. Data Analysis

#### 2.5.1. Data Preprocessing

During the data preprocessing stage, data epochs of varying sizes and targets were extracted from the raw experimental data and were subjected to downsampling and filtering. A data epoch contains data from a single stimulus cycle, which has a duration of 0.52 s and corresponds to a 31-bit code at 60 Hz.

In the training stage of the offline experiment, the data corresponding to all targets were derived from the reference target through cyclic shifting. A temporal lag denoted as $\Delta t_{k}=\frac{k}{f_{r}}$ exists between the kth and (k+1)th targets, attributed to a cyclic shift of *k* bits and a refresh rate of $f_{r}$ = 60 Hz from the reference target. This allows for the calculation of the sample point lag between targets as follows:

(6) $$\Delta \tau_{k}=floor(\Delta t_{k}\times f_{s}),k=1,2,\cdots 30$$

**Figure 4:**
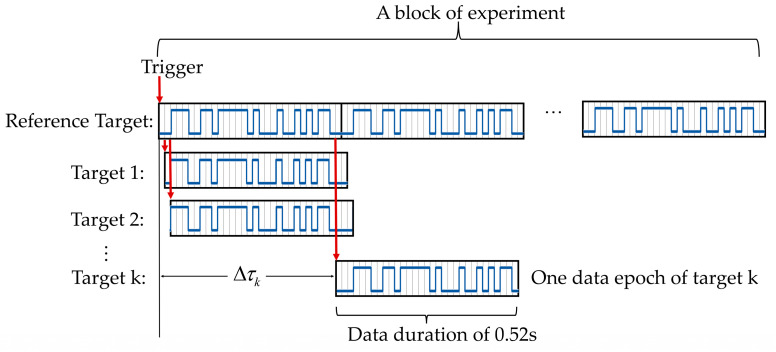
Data epoching strategy of each target from a block in the training stage of the offline experiment. A single trial of a data epoch started at a trigger after each frame of stimuli and was cyclically shifted to generate the data epoch of all targets.

Here, floor(⋅) represents rounding down to the nearest integer, *k* represents the index of the target, $\Delta t_{k}$ signifies the time lag between the kth target and the (k+1)th target, and $f_{s}$ denotes the sampling rate. In each block of the training stage, the 31-bit code was displayed sequentially for 12 stimulus cycles, allowing for the extraction of 11 epochs, as shown in Figure 4. Considering the visual onset for the subjects, the first trial of each block was discarded, resulting in 10 data epochs being collected from each block. This approach ultimately provided a total of 100 epochs of data per target across the 10 blocks of the training stage. Each data epoch derived from each block spanned 0.66 s, incorporating a visual delay of 0.14 s attributable to the conduction time of the visual pathway, and a stimulus period of 0.52 s, corresponding to 31 frames at a 60 Hz refresh rate. Before filtering, the epoch data were downsampled to 250 Hz, employing the “downsample” function in MATLAB to enhance computational efficiency. The data epoch of each trial was filtered using a fifth-order Chebyshev Type I bandpass filter with a frequency range of 3 Hz to 100 Hz, using the “cheb1ord” and “cheby1” functions in MATLAB. Finally, the visual delay was removed from these epochs and 129 data points were extracted, corresponding to the stimulus.

During the testing phase of both the offline and online experiments, data epochs were extracted for each trial based on triggers. The length of the data epoch and the method for processing the data epoch were identical to those used in the training stage. Subsequently, the visual delay was removed from these epochs and 129 data points were extracted, corresponding to the stimulus. Finally, during the testing stage of the offline experiment, each target received 7 data epochs in each trial. In the online experiment, subjects were allocated varying numbers of data epochs based on the results of the offline experiment. 

#### 2.5.2. Performance Evaluation

The offline analysis utilized data obtained from the offline experiment to assess the classification performance for 4 stimulus sizes. Ten blocks of training data were utilized for training the individualized template and TDCA spatio-temporal filters. Offline classification accuracies of the test data were computed through the TDCA method, with data lengths ranging from 0.52 s to 3.62 s (1 to 7 epochs in 1-epoch steps). Additionally, the performance of a 9-electrode layout from the 64-electrode cap (Pz, PO3, PO5, POz, PO4, PO6, O1, Oz, and O2) was also calculated to compare with the 66/256 layout. The 9-electrode layout has been widely used in previous studies [9,10,11,29]. Furthermore, a detailed analysis of the classification performance under different numbers of training blocks was conducted.

ITR is utilized to assess the performance of the system in both offline and online analyses, and is defined as follows:

(7) $$ITR=\left(\log_{2}M+P\log_{2}P+(1-P)\log_{2}\left(\frac{1-P}{M-1}\right)\right)\times \left(\frac{60}{T}\right)$$

where *M* denotes the total count of targets, *P* symbolizes the classification accuracy, and *T* represents the duration necessary for a selection, encompassing the time required for gaze transition (0.5 s in this study) and visual stimulation. Subsequently, for each stimulus size, the duration that achieved the highest ITR was selected as the data length to be used in the online experiment.

#### 2.5.3. Electrode Selection

To illustrate the benefits of high-density EEG more comprehensively, this study simulated the classification performance of three EEG caps using data from the offline experiment. The comparison of the classification performance levels achieved by three electrode configurations was performed considering two aspects: a fixed electrode coverage and a fixed number of electrodes. For the fixed electrode coverage condition, in order to ensure a uniform electrode coverage area for each step, electrodes are incrementally added in a column-wise sequence to both the left and right sides from the center of the electrode cap (POOz, Pz, POz, Oz, PPOz, CBz) for each electrode layout. The detailed sequences of electrode additions for the three layouts are listed in Table 1. For the condition with a fixed number of electrodes, a greedy algorithm was employed to seek the optimal classification results for each electrode quantity within three electrode layouts. The three electrode layouts begin with electrode quantities of 66, 32, and 21, respectively. At each step, one electrode is progressively eliminated, retaining the electrode combination that achieves the highest classification performance until the remaining electrode quantity reaches 2.

## 3. Results

### 3.1. EEG Feature

**Figure 5:**
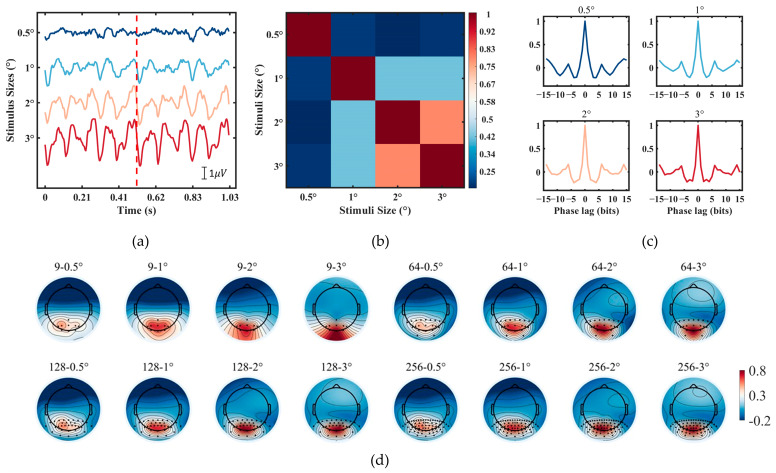
(**a**) The average EEG data of Oz across all trials from a typical subject (S5) at 0.5°(blue), 1° (light blue), 2° (yellow), and 3° (red). were divided into two trials separated by a red line. (**b**) The cross-correlation among data of the four stimulus sizes. (**c**) The autocorrelation characteristics of TDCA templates for the four stimulus sizes, averaged across all subjects. (**d**) The averaged activation pattern of the first component in TDCA across all subjects for the 9/64, 21/64, 32/128, and 66/256 layouts.

Figure 5a depicts the average EEG waveforms of two consecutive trials from a typical subject for different stimulus sizes of 0.5°, 1°, 2°, and 3°. The amplitude ranges for these waveforms are −1.87 μV to 1.07 μV, −3.64 μV to 1.87 μV, −4.40 μV to 3.40 μV, and −5.56 μV to 3.82 μV, respectively. With the increase in stimulus size, there is an augmentation in the amplitude of the EEG response, accompanied by a notable periodicity across the two trials. Figure 5b illustrates the correlation among the average waveforms for stimuli of four different sizes. It can be observed that as the stimulus size decreases, there is a reduction in its correlation with the larger stimuli, possibly due to the interference by the peripheral stimuli surrounding the attended target. Figure 5c exhibits the autocorrelation of the mean EEG data from the Oz electrode for 15 subjects at 0.5°, 1°, 2°, and 3°. Except for one with a zero lag, the autocorrelation coefficients range from −0.21 to 0.37, −0.21 to 0.26, −0.23 to 0.17, and −0.21 to 0.16, respectively. It is evident that larger stimuli exhibit superior correlation characteristics compared to the smaller stimuli, which indicates higher separability of targets. Figure 5d shows the average activation pattern of the first component in TDCA for the 9/64, 21/64, 32/128, and 66/256 layouts. With an increasing electrode density, layouts featuring denser electrodes possess more accurate spatial distributions of EEG responses, particularly for smaller stimulus sizes. Higher-density electrodes have the potential to capture a more precise spatial distribution of EEG responses, thereby enhancing the classification performance.

### 3.2. Offline Results

**Figure 6:**
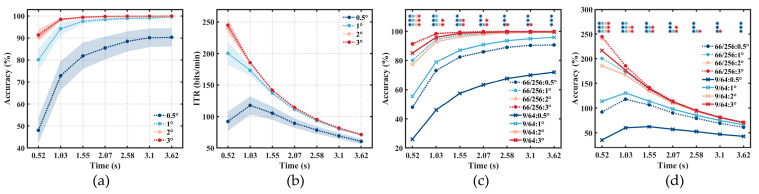
The accuracy (**a**) and ITR (**b**) were calculated using the 66/256 layout in the offline experiment. The shaded area indicates the standard errors. (**c**) The accuracy and (**d**) ITR of the 66/256 layout and the 9/64 layout. The asterisks arranged vertically indicate significant differences (* *p* < 0.05, ** *p* < 0.01, *** *p* < 0.001) between the 66/256 layout and the 9/64 layout at 0.5°(blue), 1° (light blue), 2° (yellow), and 3° (red).

The average offline accuracy and ITR of the test stage across all subjects’ three trials in the offline experiment were obtained, as depicted in Figure 6a,b. The four stimuli sizes of 0.5°, 1°, 2°, and 3° achieved accuracies of 48.06% ± 5.82%, 80.14% ± 4.90%, 90.56% ± 2.33%, and 91.46% ± 2.63%, respectively, at a stimulation duration of 0.52 s, using the 66/256 layout. As the stimulus size increased, the system’s classification performance gradually improved, peaking at 3° without a significant enhancement compared to 2° (*p* = 0.65 in 0.52 s). For the averaged ITR data across all subjects, except for the 0.5° stimulus achieving the highest ITR of 117.60 bits/min at 1.03 s, the other three sizes obtained their peak ITRs at 0.52 s, reaching 200.47 bits/min (1°), 240.30 bits/min (2°), and 244.88 bits/min (3°), respectively. Figure 6c,d further contrasts the performance levels between the 9/64 layout and the 66/256 layout. The 66/256 layout shows significantly improved classification accuracies and ITRs. Under a stimulation duration of 0.52 s, the 66/258 layout exhibits strong significance (*p* < 0.001 for four stimulus sizes) across all four stimulus sizes. Additionally, it displays strong significance (*p* < 0.001 at stimulation durations of from 0.52 s to 3.62 s) with the 0.5° stimulus, evident throughout all stimulation durations. This suggests that utilizing 256-electrode EEG caps contributes to improving system performance under shorter stimulation durations and smaller stimulus sizes.

**Figure 7:**
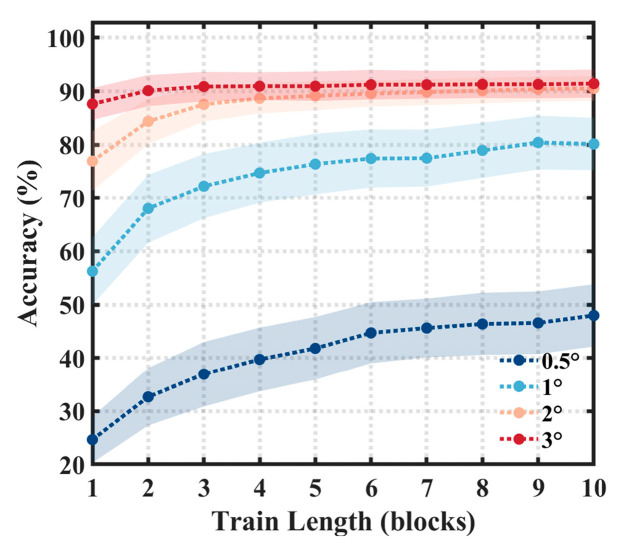
The offline accuracy with different numbers of blocks for training, using the 66/256 layout. The shaded area indicates the standard errors.

To assess the impact of the quantity of training data on the classification performance, we investigated the classification performance levels with different numbers of training blocks at a stimulation duration of 0.52 s, as shown in Figure 7. For larger stimuli (2°, 3°), the accuracy initially ascends rapidly, followed by a gradual increase before eventually stabilizing at over 90%. For smaller stimuli (0.5°, 1°), as the number of blocks increases, the performance continuously improves. The smaller the size of the stimulus, the more training blocks are needed to achieve a stable level of accuracy.

### 3.3. Online Results

Table 2 presents the data lengths utilized for each subject in the online experiment across the four stimulus sizes, along with the ITRs and accuracies achieved. Average ITRs of 126.48 ± 14.14 bits/min, 221.73 ± 15.69 bits/min, 258.39 ± 9.28 bits/min, and 266.40 ± 6.52 bits/min were obtained for 0.5°, 1°, 2°, and 3° stimuli, respectively. The online performance of the system exhibited a consistent trend with the offline predictions as the stimulus size varied. The highest ITRs achieved by individual subjects were 199.27 bits/min (S6) under the 0.5° condition, 289.59 bits/min (S4, S6, S12) under the 1° condition, 289.59 bits/min (S6, S10, S12, S16) under the 2° condition, and 289.59 bits/min (S10, S12, S16) under the 3° condition. When employing smaller stimuli, selecting online data lengths based on subjects’ offline ITRs led to higher accuracies, reaching 82.15 ± 4.18% and 93.13 ± 1.87% for 0.5° and 1° stimuli, respectively, thereby enhancing the system usability. For larger stimuli, the majority of subjects (11 subjects for 2° stimuli and 13 subjects for 3° stimuli) achieved ITRs exceeding 250 bits/min.

### 3.4. Electrode Selection Experiment

**Figure 8:**
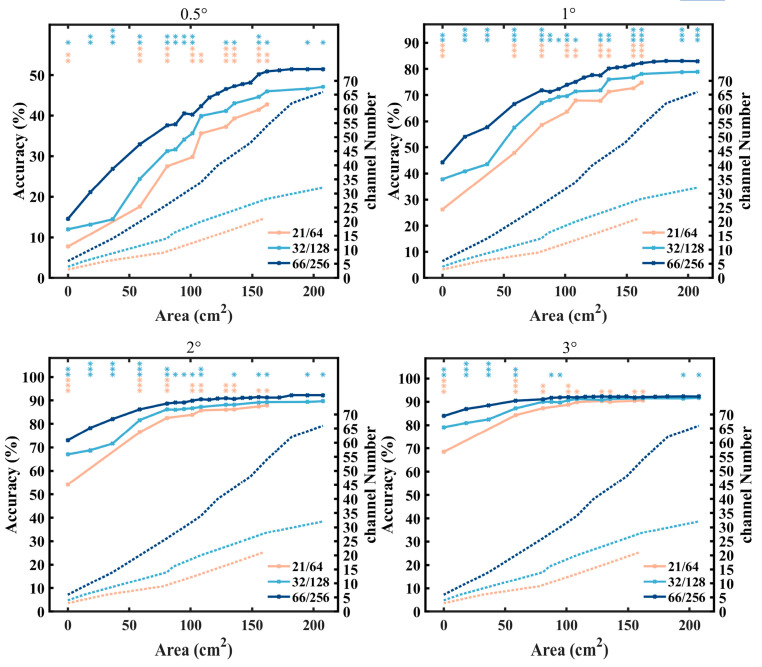
The relationship between electrode coverage area and offline accuracy. Solid lines represent the accuracy of three layouts calculated with different electrode coverage areas. Dashed lines represent the electrode number for different electrode layouts. The vertically aligned orange and blue asterisks, respectively, indicate the significant differences between the 66/256 layout and the 21/64 layout, and between the 66/256 layout and the 32/128 layout (* *p* < 0.05, ** *p* < 0.01, *** *p* < 0.001).

Figure 8 illustrates the offline classification accuracies of the three electrode layouts under different electrode coverage areas at a stimulation duration of 0.52 s. To better reflect factors influencing system performance, the electrode coverage area gradually increased from the center to both sides, ensuring that at each step, the three electrode layouts possessed an equivalent electrode coverage area. For all stimulus sizes, at equivalent coverage areas, higher-density layouts demonstrated superior classification performance levels. Under all electrode coverage areas, the 66/256 layout exhibited significant improvements compared to the 21/64 layout, with smaller stimuli exhibiting more pronounced enhancements (0.5°, 1°: *p* < 0.01; 2°, 3°: *p* < 0.05). Moreover, the 66/256 layout showed significant improvements compared to the 32/128 layout for the stimuli sizes of 0.5°, 1°, and 2° (*p* < 0.05) across all electrode coverage areas, and for the stimuli size of 3° when the coverage areas were less than 100 cm^2^ (*p* < 0.05). This finding validates that, when recording from the same region, an increase in electrode density provides richer information for detecting small stimuli.

**Figure 9:**
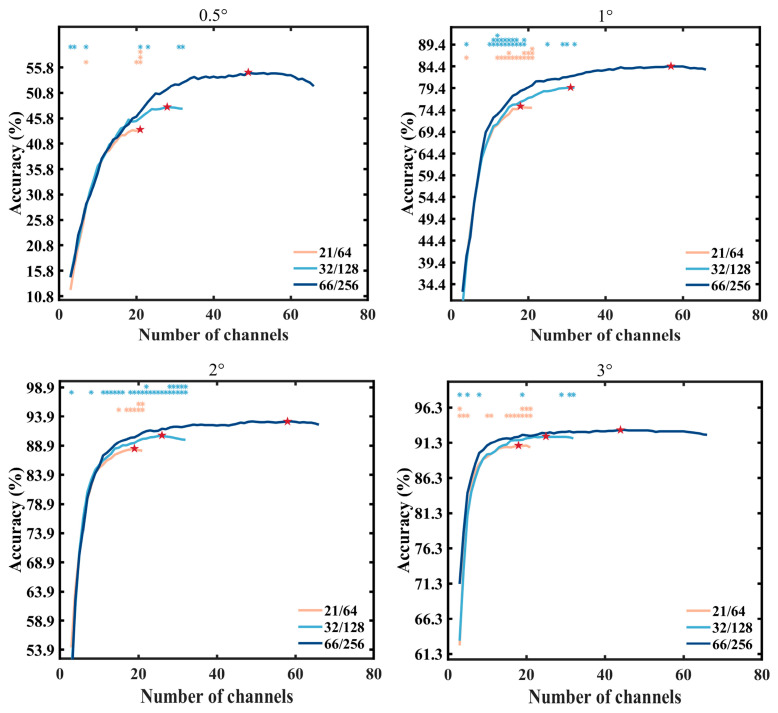
Accuracy of different electrode numbers. The vertically aligned orange and blue asterisks, respectively, indicate the significant differences between the 66/256 layout and the 21/64 layout, and between the 66/256 layout and the 32/128 layout (* *p* < 0.05, ** *p* < 0.01, *** *p* < 0.001). The red star indicates the channel number that achieved the best performance in each electrode layout.

To demonstrate the performance levels of three electrode layouts with an equal number of electrodes, a greedy algorithm was employed to calculate the optimal performance for each layout under varying electrode counts at a stimulation duration of 0.52 s, as depicted in Figure 9. Overall, for all stimulus sizes, the 66/256 layout consistently achieved the highest system performance compared to the other two layouts (under 0.5° stimulus condition, 21/64: 43.61%, 32/128: 48.06%, 66/256: 54.86%; under 1° stimulus condition, 21/64: 75.28%, 32/128: 79.58%, 66/256: 84.44%; under 2° stimulus condition, 21/64: 88.40%, 32/128: 90.69%, 66/256: 93.06%; under 3° stimulus condition, 21/64: 90.90%, 32/128: 92.22%, 66/256: 93.13%). Additionally, for smaller stimuli (0.5°, 1°), it showed a greater improvement in accuracy compared to the larger stimuli (2°, 3°). With a small number of electrodes (number of electrodes < 15), the three layouts achieved similar accuracies, and as the number of electrodes was increased, the classification performance improved rapidly. With a moderate number of electrodes (15 < number of electrodes < 30), significant differences (*p* < 0.05) among the three layouts were observed for some electrode numbers. Finally, as the number of electrodes was increased further, the system performance stabilized. It is noteworthy that the highest system performance occurred within the range of 33–41 electrodes across all three layouts (0.5°:49, 1°:57, 2°:58, 3°:44). The 66/256 layout holds a superior position, particularly evident in the case of small stimuli. This finding highlights that, when a certain number of electrodes is given, the 66/256 layout can select an electrode combination with a superior performance compared to the 32/128 layout and the 21/64 layout. However, for the classification task in this study, the 66/256 layout exhibited some redundancy.

## 4. Discussion

Previous studies have demonstrated the suitability of C-VEP for multi-target classification. The use of a pseudo-random code proves effective in generating numerous targets within a brief training period. Table 3 presents the stimulus sizes and system performance levels in recent C-VEP BCI studies [29,30,31,32,33,34]. In the studies where subjects’ distance from the screen was not specified, a commonly assumed distance of 60 cm was used for estimating the stimulus size. This study investigated EEG responses and classification performance levels across four stimulus sizes, 0.5°, 1°, 2°, and 3° and the ITRs we found were 126.48 ± 14.14 bits/min, 221.73 ± 15.69 bits/min, 258.39 ± 9.28 bits/min, and 266.40 ± 6.52 bits/min, respectively. The stimulus sizes of 1° are notably smaller than the stimulus sizes typically utilized in existing BCI systems. However, due to the utilization of a high-density EEG and the principle of equivalent neighbors in C-VEP, the BCI system proposed in this study with stimulus sizes of 1° outperforms other systems employing larger stimuli.

**Figure 10:**
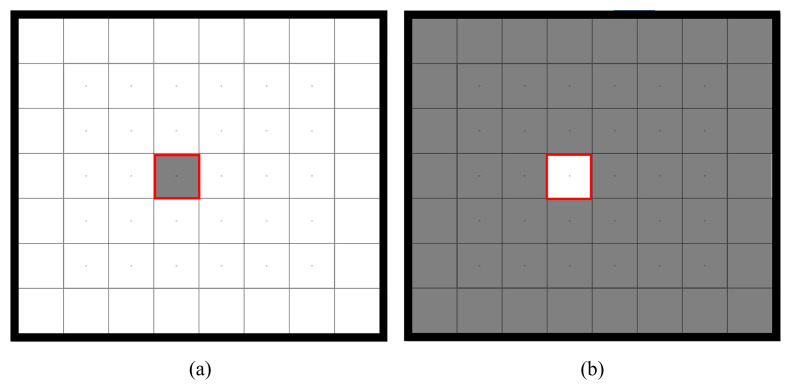
(**a**) Central and (**b**) peripheral stimulus types. The shaded regions indicate the stimulation areas and the stimulus marked with a red frame was designated as the reference target.

**Figure 11:**
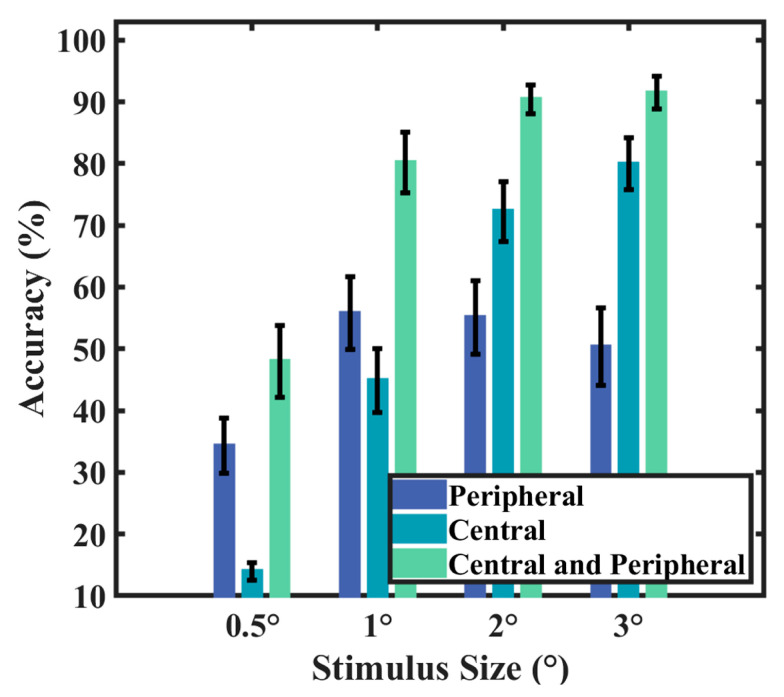
The accuracy of different stimulus types. The error bars indicate the standard errors.

Previous research indicates that the human ocular system shows increased sensitivity to light that enters through the central region of the pupil compared to light rays deviating from the center [35,36], and the response evoked by stimuli extending from the foveal area to approximately 5° of the visual angle follows a Gaussian distribution in relation to stimulus eccentricity and diminishes accordingly [37]. Therefore, when the stimulus is very small, competing stimuli can generate increased interference with the gazed stimulus. Benefiting from the principle of equivalent neighbors of C-VEP, peripheral stimuli contribute to the enhancement of the central stimulus rather than causing “interference”. It can be argued that, in the C-VEP paradigm arranged according to the principle of equivalent neighbors, the central and peripheral stimuli collectively constitute the attended target. To verify this, an additional offline experiment was conducted to clarify the unique contributions of central and peripheral stimuli to system performance. Figure 10 shows the “central”-type and “peripheral”-type stimuli used in the training stage of the offline experiment. The stimulus type described in the “Materials and Methods” section was considered to be the “central and peripheral” type. The experimental setups for the “central” type and “peripheral” type were identical to that of the “central and peripheral” type. In the “central” type, only the central stimulus marked by the red frame flickers, while the peripheral stimuli maintain a constant brightness of 255 (white). In the “peripheral” type, the central stimulus maintains a constant brightness of 255 (white), while the peripheral stimuli are allowed to play out normally. Individualized templates and TDCA spatio-temporal filters were created using both stimulus types. Testing was conducted using the test data from the “Offline Experiment” section. As shown in Figure 11, all four stimuli sizes achieved the highest performance under the “central and peripheral” type, with the worst under the “central” condition with stimulus sizes below 2°. This suggests that, in the case of small stimuli, peripheral stimuli contribute to a certain level of classification performance. Therefore, during the training stage, it is necessary to take peripheral stimuli into consideration.

In recent years, many studies have confirmed the need to increase the density of electrodes to improve the spatial resolution of an EEG. Compared to sampling an EEG with a 3 cm inter-electrode distance, the spatial sampling of functional brain signals with a 1 cm inter-electrode distance resulted in an approximately twofold increase in the signal-to-noise ratio [23]. Electrodes with a higher density were found capable of detecting more complex neural activation patterns [24]. The findings of this study are consistent with those of previous research. Higher-density electrodes achieve a superior classification performance, as illustrated in Figure 8 and Figure 9. In addition, as shown in Figure 5d, high-density electrodes acquire richer spatial patterns, especially for small stimuli that may activate neural activity with a higher spatial density in the visual cortex. In the literature, Zelmann et al. simulated distributed sources of approximately 1 cm^2^ and employed the 10-20 system, the 10-10 system, and 10-5 system [38]. They found that the small sources were visible on the scalp at 14%, 38%, and 71%, respectively. This suggests that by directly placing electrodes above these focal generators, high-frequency oscillations with lower amplitudes and high localization can be captured on the scalp. The small stimuli may activate a smaller area of the visual cortex and evoke weaker EEG responses. In this case, high-density electrodes have a higher probability of recording these responses. Figure 9 shows that a high-density electrode layout (66/256 layout) consistently exhibits a superior classification performance with different numbers of electrodes, indicating that high-density electrodes are more adept at obtaining electrode combinations with a better signal quality. With the advancement of high-density electrode technology, the classification performance levels with further increased electrode densities will be investigated in our future work.

Recently, many studies have been dedicated to developing BCI systems with a large number of targets [12,39,40]. For example, a group modulation method can encode a large number of targets (over 100 targets) on a C-VEP BCI [29]. When using small stimuli, more targets can be placed in the same stimulus area, making the GUI more suitable for daily use scenarios. Using the group encoding method with small stimuli, the C-VEP BCI system can enable the display of a large number of targets on screens. For example, more than 600 1° stimuli can be rendered on a 24-inch monitor. Furthermore, large stimuli inherently imply a limited number of targets due to the small screen area. In the near future, with the advancement of portable devices like mobile phones, there is a clear anticipation that BCI systems with small stimuli will be employed on compact devices to broaden the scope of their application scenarios.

## 5. Conclusions

In this study, a high-density 256-electrode EEG cap was employed to assess the signal characteristics and classification performance of 30-class C-VEP signals across four stimulus sizes (0.5°, 1°, 2°, and 3°). The results indicate that the application of a high-density EEG cap significantly enhances the performance of C-VEP BCIs. Notably, by optimizing the data length used in online experiments through offline experiments, ITRs of 126.48 ± 14.14 bits/min, 221.73 ± 15.69 bits/min, 258.39 ± 9.28 bits/min, and 266.40 ± 6.52 bits/min were achieved for 0.5°, 1°, 2°, and 3° stimuli, respectively. Moreover, the results suggest that it is crucial to consider the contribution of a high-density EEG when utilizing small stimuli. These findings provide useful guidelines for designing and implementing high-performance C-VEP BCIs with small stimuli.

## Figures and Tables

**Table 1:** The detailed sequence of electrode additions for the three layouts.

Order of Columns	Columns	21/64 Layout	32/128 Layout	66/256 Layout	Area (cm^2^)
Initial	Z	POz, Oz, Pz	POz, Oz, Pz, CBz	POOz, Pz, POz, Oz, PPOz, CBz	0
1	1h/2h	―	PPO1h, PPO2h	OCB1h, O1h, P1h, PPO1h, OCB2h, O2h, P2h, PPO2h	35.99
2	1/2	P1, P2, O1, O2, CB1, CB2	PO1, PO2, P1, P2, O1, O2, CB1, CB2	POO1, P1, O1, CB1, OCB1, PO1, OCB2, P2, O2, CB2, POO2, PO2	79.61
3	3h/4h	―	PPO3h, PPO4h	P3h, PPO3h, P4h, PPO4h	93.35
4	3/4	PO3, PO4, P3, P4	PO3, PO4, P3, P4	PO3, PO4, P3, P4	106.89
5	5h/6h	―	―	P5h, POO5h, PPO5h, P6h, POO6h, PPO6h	120.31
6	5/6	PO5, PO6, P5, P6	PO5, PO6, P5, P6	PO5, PO6, P5, P6	133.55
7	7h/8h	―	―	PPO7h, P7h, PPO8h, P8h	146.70
8	7/8	PO7, PO8, P7, P8	PO7, PO8, P7, P8	P7, PO7, POO7, P8, PO8, POO8	159.86
9	9h/10h	―	―	P9h, PO9h, PPO9h, POO9h, P10h, PO10h, PPO10h, POO10h	179.74
10	9/10	―	PO9, PO10, P9, P10	PO9, PO10, P9, P10	204.75

**Table 2:** The data trials, accuracy, and ITR obtained for each stimulus size in the online experiment.

Subjects	0.5°			1°			2°			3°		
	Data Trials	Accuracy (%)	ITR (bits/min)	Data Trials	Accuracy (%)	ITR (bits/min)	Data Trials	Accuracy (%)	ITR (bits/min)	Data Trials	Accuracy (%)	ITR (bits/min)
S1	3	91.11	118.38	2	90	154.88	2	100	192.01	1	95.56	262.94
S2	2	95.56	173.45	1	95.56	261.60	1	94.44	257.48	1	95.56	262.94
S3	3	87.78	110.61	2	100	192.01	1	92.22	245.04	1	93.33	250.15
S4	2	100	192.01	1	100	289.59	1	98.89	282.25	1	98.89	282.25
S5	2	86.67	144.68	1	95.56	262.94	1	97.78	276.26	1	98.89	282.25
S6	1	82.22	199.27	1	100	289.59	1	100	289.59	1	97.78	276.26
S7	3	68.89	73.91	1	71.11	155.94	1	84.44	208.77	1	92.22	245.04
S8	5	36.67	17.21	2	95.56	173.45	1	90	236.99	1	93.33	249.62
S9	6	66.67	40.30	3	83.33	101.26	2	95.56	174.69	2	98.89	187.15
S10	2	100	192.01	1	97.78	274.92	1	100	289.59	1	100	289.59
S11	3	83.33	101.01	1	94.44	255.61	1	98.89	282.25	1	98.89	282.25
S12	2	100	192.01	1	100	289.59	1	100	289.59	1	100	289.59
S13	2	77.78	120.15	1	93.33	250.15	1	96.67	268.93	1	94.44	255.61
S14	3	78.89	91.96	3	92.22	121.53	1	96.67	268.93	1	97.78	274.92
S15	2	93.33	166.45	1	88.89	229.65	1	98.89	268.93	1	98.89	282.25
S16	2	65.56	90.39	1	92.22	245.04	1	100	289.59	1	100	289.59
Mean	-	82.15	126.48	-	93.13	221.73	-	96.52	258.39	-	97.15	266.40
STE	-	4.18	14.14	-	1.87	15.69	-	1.10	9.28	-	0.67	6.52

**Table 3:** The performance and stimuli size in recent C-VEP BCI studies.

Article	Number of Targets	Stimuli Size	Accuracy (%)	ITR (bits/min)
Wei et al., 2018 [29]	48	2.64°	91.67	181.05
Başaklar et al., 2019 [30]	36	4.95°	97	94.21
Gembler et al., 2019 [31]	32	6.2°	100	149.3
Gembler et al., 2020 [32]	32	4.04°	95.5	96.9
Zarei et al., 2022 [33]	32	3.8°	88.13	187.38
Fernández-Rodrígue et al., 2023 [34]	9	6.7°	96.53	164.54
This study	30	1°	93.13	221.73
This study	30	3°	97.15	266.40

## Data Availability

The data presented in this study are openly available on FigShare at https://dx.doi.org/10.6084/m9.figshare.25714260 (accessed on 30 April 2024). We have ensured that the data shared are in accordance with the consent provided by the participants on the use of confidential data.
